# Identification of the Prognostic Signature Associated With Tumor Immune Microenvironment of Uterine Corpus Endometrial Carcinoma Based on Ferroptosis-Related Genes

**DOI:** 10.3389/fcell.2021.735013

**Published:** 2021-10-06

**Authors:** Jinhui Liu, Yichun Wang, Huangyang Meng, Yin Yin, Hongjun Zhu, Tingting Ni

**Affiliations:** ^1^Department of Gynecology, The First Affiliated Hospital of Nanjing Medical University, Nanjing, China; ^2^Department of Urology, The First Affiliated Hospital of Nanjing Medical University, Nanjing, China; ^3^Department of Obstetrics and Gynecology, The First Affiliated Hospital of Nanjing Medical University, Nanjing, China; ^4^Department of Oncology, Nantong Third People’s Hospital Affiliated to Nantong University, Nantong, China; ^5^Department of Oncology, Affiliated Tumor Hospital to Nantong University, Nantong, China

**Keywords:** uterine corpus endometrial carcinoma, ferroptosis, prognostic signature, molecular subtypes, immune checkpoint, drug sensitivity

## Abstract

**Background:** Uterine corpus endometrial carcinoma (UCEC) is the sixth most common cancer worldwide. Ferroptosis plays an important role in malignant tumors. However, the study of ferroptosis in the endometrial carcinoma remains blank.

**Methods:** First, we constructed a ferroptosis-related signature based on the expression profiles from The Cancer Genome Atlas database. Then, patients were divided into the high-risk and low-risk groups based on this signature. The signature was evaluated by Kaplan–Meier analysis and receiver operating characteristic (ROC) analysis. We further investigated the relationship between this signature and immune microenvironment via CIBERSORT algorithm, ImmuCellAI, MAF, MSI sensor algorithm, GSEA, and GDSC.

**Results:** This signature could be an independent prognostic factor based on multivariate Cox regression analysis. GSEA revealed that this signature was associated with immune-related phenotype. In addition, we indicated the different status of immune infiltration and response to the immune checkpoint between low-risk and high-risk groups. Patients in the low-risk group were more likely to present with a higher expression of immune checkpoint molecules and tumor mutation burden. Meanwhile, the low-risk patients showed sensitive responses to chemotherapy drugs.

**Conclusion:** In summary, the six ferroptosis-related genes signature could be used in molecular subgrouping and accurately predict the prognosis of UCEC.

## Introduction

Uterine corpus endometrial carcinoma (UCEC) is a huge threat to women’s health, whose incidence is increasing year by year in the United States (Siegel et al., 2019). Most women diagnosed with highly differentiated endometrial histology tend to be diagnosed early and have a favorable prognosis (Amant et al., 2005). However, some patients have low-grade, early stage, well-differentiated endometrioid tumors, in which unexpected recurrence and poor prognosis have indeed occurred. For patients with relapsed or advanced tumors and clinically aggressive histological tumors, the clinical outcome will be greatly worsened (Siegel et al., 2018). Such a poor prognosis of endometrial cancer highlights the urgent need to understand the mechanism of tumorigenesis and develop more effective strategies for predicting patients’ prognosis.

Ferroptosis is an iron-dependent regulation of cell death mediated by the fatal accumulation of lipid peroxides (Dixon et al., 2012). Artificial introduction of ferroptosis is considered a promising treatment for cancers resistant to traditional therapies (Hassannia et al., 2019; Liang et al., 2019). Ferroptosis has been reported to be a crucial process in human hepatocellular carcinoma, and CDGSH iron sulfur domain 1 (CISD1) (Yuan et al., 2016) and TP53 gene are known to be negative regulatory effective for ferroptosis (Jennis et al., 2016). In addition, other genes such as retinoblastoma (Rb), nuclear factor erythroid 2–related factor 2 (NRF2), and metallothionein (MT)-1G are reported to be associated with ferroptosis and protect liver cancer from induction of sorafenib (Louandre et al., 2015; Sun et al., 2016a,b). In 2021, researchers have revealed that ferroptosis process was aberrantly regulated in UCEC and an activator of ferroptosis can induce cell death in UCEC cells (Lopez-Janeiro et al., 2021; Zhang et al., 2021). However, the prognosis value of ferroptosis in the endometrial carcinoma still remains blank.

In the present study, expression profiles and clinical data of 511 UCEC patients from The Cancer Genome Atlas (TCGA) were used. We developed a ferroptosis-related prognostic signature. The prognostic role of the ferroptosis-related prognosis signature (FRPS) was identified by multi-faceted analysis. The relationships between the signature and immune cell type fractions, immune checkpoint modulators, mutation profile, consensus clustering, m6A regulators, mRNAsi, and functional analyses were further evaluated to explore underlying value of the FRPS.

## Materials and Methods

### Data Collection

All these expression profiles and corresponding clinical data were obtained from TCGA^1^. Then, complete clinical data of 548 UCEC samples and 23 normal samples including survival time were filtered for further analysis. We integrated the transcriptome and complete clinical data to screen out 511 overall survival–related UCEC samples. Half of them (*n* = 256) were randomly split into training cohort. The entire patients (*n* = 511) were defined as testing cohort to verify the signature. The baseline information is exhibited in Supplementary Table 1. Then, 60 ferroptosis-related genes were retrieved from the gene list provided by previous literature (Liang et al., 2020). A total of 15 UCEC specimens and 15 adjacent tissues were obtained from the Affiliated Tumor Hospital of Nantong University. We obtained all the written informed consent from patients.

### Development and Validation of Ferroptosis-Related Prognosis Signature

Firstly, we performed univariate Cox regression analysis to screen targeted ferroptosis-related genes with prognostic values. To reduce the risk of over-fitting, Lasso regression analysis, and univariate and multivariate Cox regression analysis were used to construct the prognosis model (Tibshirani, 1997). Lasso algorithm was used to select variables, and “glmnet” R package was used to shrink (Simon et al., 2011). The risk score of the FRPS was calculated according to the normalized expression level of each gene and its corresponding regression coefficient. The formula was FRPS risk score = Σ(the expression amount of each gene multiplied by the corresponding coefficient). According to the median risk score of the FRPS, we divided the patients into two groups. Then, principal component analysis (PCA) was performed by using “scatterplot3d” R package on the base of expression. The “survminer” R package was used for survival analysis of each gene. The R package “survival ROC” was used to evaluate the predictive ability of the signature.

### Quantitative Real-Time-PCR

Total RNA from 15 UCEC samples and 15 adjacent tissues was extracted using TRIzol reagent (Invitrogen). The residual genomic DNA from total RNA was removed by 4 × gDNA wiper Mix (Vazyme R323-01). The complementary RNA was synthesized using PrimeScript RT reagent kit. The SYBR Premix Ex Taq Kit (TaKaRa DRR041) was used to perform real-time quantification. The relative expression levels of target genes were normalized by GAPDH and estimated using the 2^−ΔΔ*Ct*^ method. The primers used in this research are listed in Supplementary Table 2.

### Establishing and Validating a Nomogram for Prognosis Prediction

Nomogram involving prognostic clinicopathological factors (age, stage, histological type, grade, and FRPS) was carried out for prognosis prediction. In validation, we used the calibration plots for calibration of the nomogram. The “rms,” “foreign,” and “survival” package in R were used to establish and validate a nomogram (Park, 2018).

### Assessment of Immune Cell Infiltration in Tumors

CIBERSORT algorithm was used to obtain the fraction of 22 immune cell types based on RNA-Seq data (Newman et al., 2015). The correlation between FRPS and these immune cells was analyzed by Spearman.

### Immunotherapy Response Prediction

We used an online tool Immune Cell Abundance Identifier (ImmuCellAI) to estimate the abundance of 24 immune cells in UCEC (Miao et al., 2020). The datasets including RNA-Seq and microarray data were used to predict the patient’s response to an existing immune checkpoint blockade therapy.

### mRNAsi, Mutation Analysis, and Functional Enrichment Analysis

The results of mRNAsi in TCGA-UCEC were obtained from a previous study (Malta et al., 2018). The mutation data of UCEC patients were downloaded from TCGA. The mutation annotation format (MAF) and MAF tool helped us to obtain somatic variation data (Mayakonda et al., 2018). The tumor mutation burden (TMB) score was obtained as follows: TMB = (total mutant bases/total covered bases) × 10^6^ (Robinson et al., 2017). The functional enrichment analysis was conducted by single-sample gene set enrichment analysis (ssGSEA) using the infiltrating score of 16 immune cells and the activity of 13 immune-related pathways.

### Microsatellite Instability Analysis

Microsatellite instability sensor algorithm is a program that can report the percentage of unstable microsatellites (Niu et al., 2014). We used this algorithm to obtain the MSI status for all cases based on somatic mutation data downloaded from TCGA. The relationship between FRPS and MSI was analyzed using Spearman’s rank correlation coefficient.

### Gene Set Enrichment Analysis

Gene set enrichment analysis is a method used to determine whether a set of marker genes can predict a statistically significant difference between two different cohorts. Here, we analyzed the significant difference in survival between the two cohorts in the entire TCGA cohort divided by the risk score. Normalized *p*-values less than 0.05 and false discovery rate (FDR) less than 0.05 are considered significantly enriched (Canzler and Hackermuller, 2020).

### Prediction of Chemotherapy Response

To evaluate the response to chemotherapy drugs, we used public pharmacogenomics database Genomics of Drug Sensitivity in Cancer (GDSC)^2^. The half-maximal inhibitory concentration (IC_50_) is estimated by R package “pRRophetic” (Yang et al., 2013).

### Consensus Clustering and Survival Analysis

To identify the molecular subtypes in endometrial cancer, the TCGA UCEC cohort was divided into different groups by R package “Consensus Cluster Plus, 1000 iterations and resampling rate of 80%” (Yu et al., 2015). We performed the log-rank test and Kaplan–Meier curve to assess the overall survival (OS) difference between different groups. Chi-square test was a good assistant helping us to compare the distribution of age, grade, stage, and histologic type between different clusters.

### Statistical Analysis

All statistics and figures were analyzed using R 3.6.2. Wilcoxon’s test allowed us to evaluate the differential expression of genes related to ferroptosis between UCEC patients and controls. We used the χ^2^ test to assess the relationship between FRPS and clinicopathological factors. *P*-value < 0.05 was considered to be statistically significant.

## Results

### Construction of Ferroptosis-Related Prognosis Signature in the Cancer Genome Atlas Training Cohort

**FIGURE 1:**
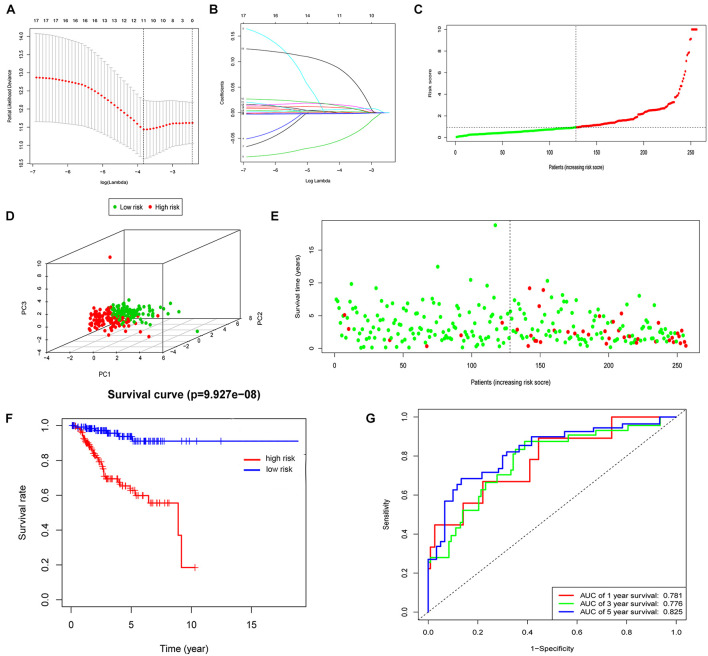
Construction of the FRPS. **(A)** A cross-validation for tuning parameter selection in the LASSO model. **(B)** LASSO coefficient profiles of 17 prognostic immune-related genes. **(C–E)** The distribution of risk score, PCA and survival status in training set. **(F)** Kaplan-Meier survival curves of overall survival between high-risk and low-risk patients in training set. **(G)** 1-year, 3-year, and 5-year ROC curve of the predictive power of the FRPS in training set.

First, the expression profiles and survival data of UCEC patients in training cohort were filtered based on 60 ferroptosis-related genes through the univariate Cox regression analysis and 17 genes were correlated with overall survival (Table 1). Then, LASSO analysis (Figures 1A,B), and univariate and multivariate Cox regression analysis narrowed the screened scope to six genes (HMOX1, KEAP1, HSBP1, SAT1, CISD1, and GPX4) (Supplementary Material). The risk score of FRPS for OS = (0.002907 × HMOX1) + (0.013486 × KEAP1) + (−0.089640 × HSBP1) + (−0.001665 × SAT1) + (0.148239 × CISD1) + (−0.003060 × GPX4). Meanwhile, patients in the training cohort were divided into high-risk and low-risk groups according to the median risk score of the FRPS (Figure 1C). PCA indicated the patients in different risk groups were distributed in two directions (Figure 1D). The distribution of FRPS and survival status of patients in OS signature are shown in Figure 1E. The OS of patients in the low-risk group was significantly longer than those in the high-risk group according to the Kaplan–Meier curve (Figure 1F). Besides, the 1-year, 3-year, and 5-year ROC curves based on training cohort are plotted as Figure 1G, suggesting satisfactory prognostic value of the signature.

**TABLE 1:** The 17 significant prognostic genes revealed by univariate Cox regression.

Gene	Hazard ratio	95% CI	*P*-value
HMOX1	1.003	1.001–1.004	0.000
GOT1	1.023	1.010–1.037	0.001
SAT1	0.997	0.995–0.999	0.001
HSBP1	0.906	0.851–0.966	0.002
ATP5MC3	1.032	1.011–1.055	0.004
KEAP1	1.018	1.005–1.031	0.006
GPX4	0.995	0.991–0.999	0.007
AKR1C3	1.006	1.001–1.011	0.015
CISD1	1.096	1.017–1.182	0.016
ACSF2	1.067	1.012–1.124	0.017
CHAC1	1.028	1.003–1.053	0.025
GCLM	1.058	1.007–1.112	0.026
GCLC	1.034	1.003–1.067	0.031
AKR1C1	1.021	1.001–1.041	0.038
CBS	2.544	1.050–6.165	0.039
CS	1.038	1.001–1.076	0.042
HSPB1	0.999	0.998–1.000	0.047

**FIGURE 2:**
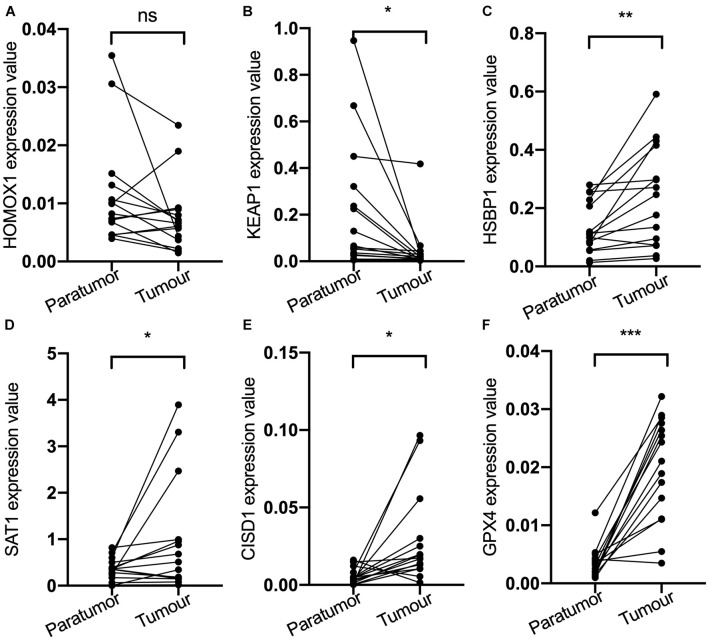
**(A)** HOMOX1 expression level. **(B)** KEAP1 expression level. **(C)** HSBP1 expression level. **(D)** SAT1 expression level. **(E)** CIDS1 expression level. **(F)** GPX4 expression level. ns, not significant, *P < 0.05, **P < 0.01, ***P < 0.001.

Then, the mRNA expression of these genes was validated by qPCR using the samples from the Nantong Third People’s Hospital Affiliated to Nantong University (Figure 2). The mRNA expression of KEAP1, HSBP1, SAT1, CISD1, and GPX4 were significantly different between tumor and the adjacent tissues. KEAP1 and HOMOX1 were low expressed in tumor than the para-carcinoma tissues, and the high expression of these two genes increased the risk of FRPS, which suggested the poor prognosis of patients. These results suggested the potential feasibility of this signature for clinical usage. Expression and Kaplan–Meier survival analysis of each gene in the signature were also performed and four genes were output significantly (Supplementary Figure 1).

### Validation of Ferroptosis-Related Prognosis Signature in the Cancer Genome Atlas Testing Cohort

**FIGURE 3:**
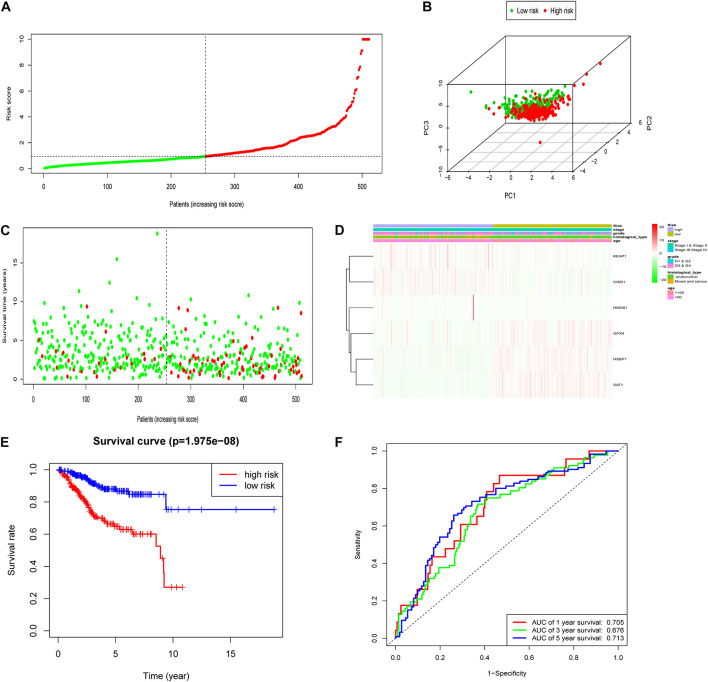
Construction of the FRPS in the testing cohort. **(A)** The distribution of the risk score among patients. **(B)** PCA analysis confirmed that patients in two subgroups were distributed in discrete directions. **(C)** The distribution of the survival status among patients. **(D)** The distribution of stage, grade, histological type, age, and the expression of six genes in high-risk and low-risk groups. **(E)** Kaplan–Meier survival curves of overall survival between high-risk and low-risk patients in testing set. **(F)** 1-year, 3-year, and 5-year ROC curve of the predictive power of the FRPS in testing set.

**FIGURE 4:**
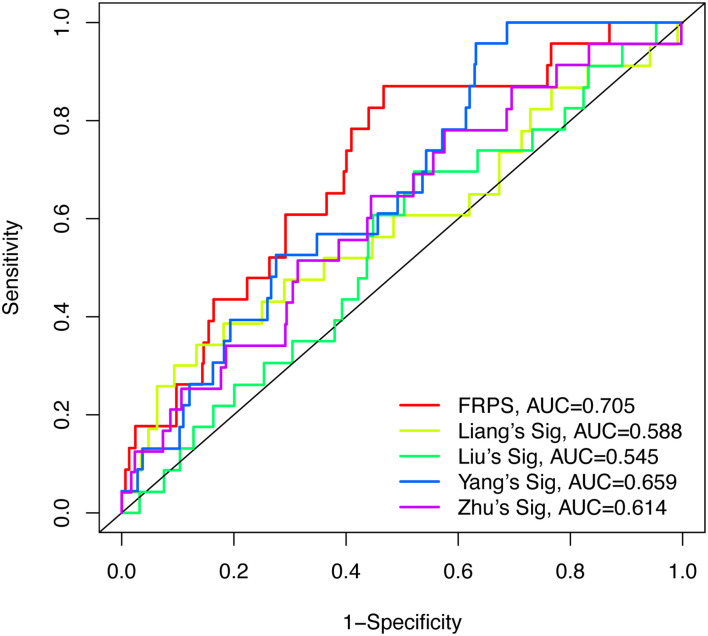
The AUC for FRPS and the existing ferroptosis-related signatures.

To test the robustness of the aforementioned signature, the entire TCGA patients were divided into high-risk and low-risk groups by the same risk score (Figure 3A). PCA confirmed the similar results obtained from the training cohort; the patients in the two subgroups were distributed in discrete directions (Figure 3B). Similarly, patients in the high-risk group showed worse prognosis (Figure 3C). The expression of six genes in the signature are exhibited in Figure 3D. Kaplan–Meier survival analysis claimed the reduced survival time of patients in the high-risk group compared with those in the low-risk group (Figure 3E). Furthermore, subgroup analyses in age, grade, stage, and histological type in the signature were performed, demonstrating that patients with high-risk scores shared shorter OS in all the subgroups (*p* < 0.05) (Supplementary Figure 2). Besides, the 1-year, 3-year, and 5-year AUC of signature was 0.705, 0.676, and 0.713, respectively (Figure 3F). According to the Cox regression analysis, the histological type, tumor stage, and this signature were independent prognostic factors (Table 2). We further compared the prediction value of this signature with other existing signatures. As shown in Figure 4, the AUC for this signature was 0.705, which was higher than the existing ferroptosis-related signatures. This result revealed that our established signature was superior to other signatures in predicting patient’s survival information (Yu et al., 2015; Liu et al., 2021; Yang et al., 2021; Zhu et al., 2021).

**TABLE 2:** Univariate and multivariate Cox regression analysis of the clinical factors and overall survival in different patient sets.

Variables	Univariable model			Multivariable mode		
		
	HR	95% CI	*P*-value	HR	95% CI	*P*-value
Train set						
Age	1.875	0.941–3.738	0.074			
Histological type	3.849	2.081–7.117	<0.001	2.665	1.377–5.160	0.004
Grade	4.237	1.024–17.542	0.046	1.645	0.367–7.370	0.515
Stage	4.714	2.540–8.746	<0.001	3.034	1.551–5.933	0.001
FRPS	1.029	1.017–1.042	<0.001	1.027	1.014–1.040	<0.001
Entire set						
Age	1.778	1.112–2.843	0.016	1.695	1.031–2.788	0.038
Histological type	3.044	2.003–4.624	<0.001	1.748	1.104–2.768	0.017
Grade	3.363	1.467–7.710	0.004	1.509	0.622–3.660	0.362
Stage	4.116	2.700–6.275	<0.001	3.286	2.078–5.194	<0.001
FRPS	1.024	1.014–1.034	<0.001	1.022	1.011–1.033	<0.001

### Establishment of Nomogram

**FIGURE 5:**
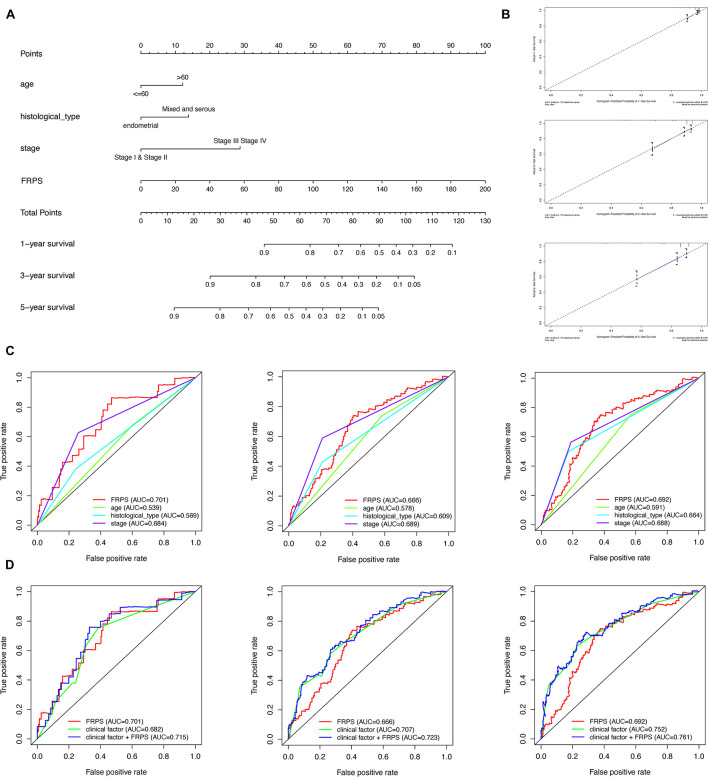
Construction and validation of the nomogram. **(A)** Nomogram to predict the probability of 1-, 3-, and 5-year OS of UCEC patients. **(B)** Calibration curves of the nomogram to predict the probability of OS at 1, 3, and 5 years. **(C)** 1-, 3-, and 5-year ROC of FRPS and the other clinical characteristics. **(D)** 1-, 3-, and 5-year ROC of the combination of FRPS and clinical factors.

To better predict overall survival time, we integrated clinicopathological factors related to prognosis (age, stage, histological type, and FRPS) to establish a nomogram prediction model (Figure 5A). We compared the relationship between FRPS and clinicopathological factors (Supplementary Table 3). Quantifying the aforementioned variables as numerical points, 1-year, 3-year, and 5-year survival rates of UCEC patients can be calculated based on the total points of all risk factors. A calibration chart was also constructed to show the consensus of the predicted and observed results (Figure 5B). Meanwhile, ROC curve demonstrated the better predictive ability of FRPS in 1-year, 3-year, and 5-year OS than other clinical factors (Figure 5C). Combining clinical factors and FRPS accessed optimal predicting effect in UCEC patients based on 1-year, 3-year, and 5-year OS (Figure 5D).

### Ferroptosis-Related Prognosis Signature and Immune Cell Type Fractions

**FIGURE 6:**
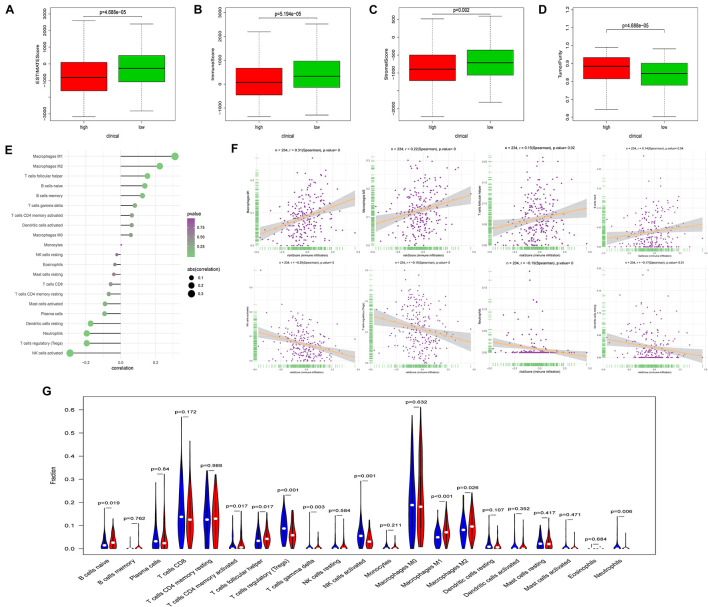
Correlation between FRPS and immune cell infiltration. **(A–D)** The correlation between FRPS and **(A)** ESTIMATE Score, **(B)** Immune Score, **(C)** Stromal Score, and **(D)** Tumor Purity. **(E)** The association between FRPS and immune cell infiltration. **(F)** The association between IRPS and each type of immune cell. **(G)** The landscape of immune cell infiltration in low-risk and high-risk groups. The low-risk and high-risk groups are represented via blue and red violin, respectively.

Using ESTIMATE algorithm, patients in the high-risk group were found to have lower immune scores, stromal scores, and ESTIMATE scores (Figures 6A–C). On the contrary, patients accessed higher tumor purity scores in the high-risk group (Figure 6D). The aforementioned findings suggested that the tumor immune microenvironment was closely associated with the FRPS in UCEC patients. To find the major immune cells between the high-risk groups and low-risk groups, CIBERSORT algorithm was employed. The results showed that macrophages M1, macrophages M2, T cell follicular helper, and B cells naive were positively correlated with FRPS while NK cells activated, T cells regulatory (Tregs), neutrophils, and dendritic cells resting were negatively correlated with FRPS (Figure 6E). The distribution of immune cells and scores for each patient is exhibited in Figure 6F. In the present research, we also focused on the tumor infiltrating cells between subgroups. We found that B cells naive, macrophages M1, macrophages M2, T cells CD4 memory activated, T cells follicular helper, T cells gamma delta, NK cells activated, T cells regulatory (Tregs), and neutrophils infiltrated differently in different groups (Figure 6G).

### Ferroptosis-Related Prognosis Signature and Immune Checkpoint Modulators

**FIGURE 7:**
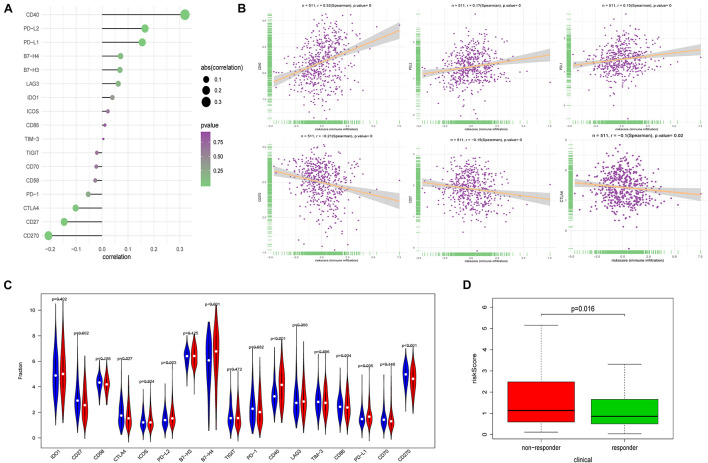
Correlation between FRPS and immune checkpoint molecules and the predicted response to immunotherapy. **(A)** The association between FRPS and immune checkpoint molecules. **(B)** The association between FRPS and several immune checkpoint molecules in detail. **(C)** The landscape of the expression of immune checkpoint molecules in low-risk and high-risk groups. **(D)** The different immunotherapy response rates in low-risk and high-risk groups.

Immune checkpoint proteins, playing important roles in the immune response, aroused our great interest to explore the relationship between FRPS and immune checkpoint modulators. The results demonstrated that CD40, PD-L1, and PD-L2 showed a positive correlation with FRPS while CD270, CD27, and CTLA4 were negatively related to FRPS (Figure 7A). The distribution of immune checkpoint proteins and risk scores for each patient is exhibited in Figure 7B. Immune checkpoint proteins between high-risk groups and low-risk groups were evaluated, and results demonstrated that CD27, CTLA4, PD-L2, B7-H4, CD40, PD-L1, and CD270 expressed differently between high- and low-risk groups (Figure 7C). Potential response to immunotherapy in each patient was further assessed by the online tool ImmuCellAI and patients in the low-risk group showed better reactivity to immunotherapy than those in the high-risk group (*p* = 0.016; Figure 7D).

### Ferroptosis-Related Prognosis Signature and Mutation Profile

**FIGURE 8:**
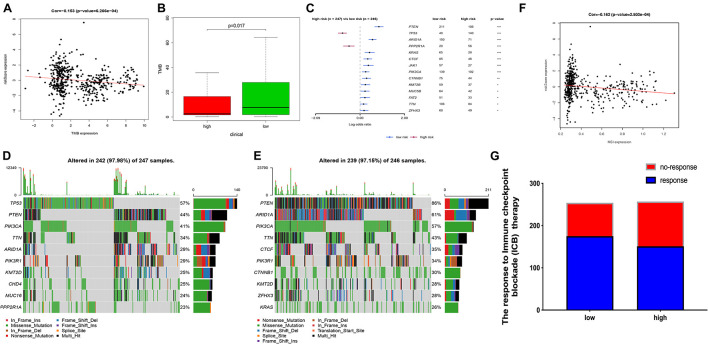
The TMB, mutation profile, and MSI in high-risk and low-risk groups. **(A)** The correlation between TMB and FRPS. **(B)** The TMB status in high-risk and low-risk groups. **(C)** The most frequently mutated genes in high-risk and low-risk groups. **(D,E)** The top 10 mutated genes in high-risk and low-risk groups. **(F)** The relationship between MSI and FRPS. **(G)** The rate of response to immunotherapy in high-risk and low-risk groups. Red line to provide a brief tendency of association between TMB, MIS and riskscore. *P < 0.05, **P < 0.01, ***P < 0.001.

Tumor mutation burden (TMB) is an important cause of tumor occurrence and development, can be used to predict the efficacy of immune checkpoint blockade, and has been shown to be a biomarker for patients who benefit from immunotherapy. In this study, we declared that FRPS was negatively correlated with TMB (Figure 8A). Lower TMB was observed in the high-risk group (Figure 8B). In addition, the mutant genes that showed the most significant difference in their mutation frequency between the two groups are shown in Figure 8C. TP53 and PPP2R1A were found to have higher mutation frequency in the high-risk group, and the rest of the genes showed higher mutation frequency in the low-risk group. Therefore, somatic mutation data were used to assess the TMB of patients. The order of somatic mutation frequency in the high-risk group was TP53 > PTEN > PIK3CA > TTN > ARID1A > PIK3R1 > KMT2D > CHD4 > MUC16 > PPP2R1A (Figure 8D); in the low-risk group, PTEN > ARID1A > PIK3CA > TTN > CTCF > PIK3R1 > CTNNB1 > KMT2D > ZFHX3 > KRAS (Figure 8E).

### Ferroptosis-Related Prognosis Signature and Microsatellite Instability

Several researches had illustrated that MSI can affect the effect of immunotherapy in several cancers. In this research, we also investigated the MSI status between groups. The results revealed that MSI status was negatively correlated with FRPS (Figure 8F). Besides, according to ImmuCellAI, higher immunotherapy response rate was observed in the low-risk group compared with patients in the high-risk group (Figure 8G), which implied that patients in the low-risk group might benefit from immunotherapy.

### m6A Regulators, mRNAsi, and Functional Analyses in Two Groups

In recent years, the role of m6A methylation in cancer has attracted widespread attention. More and more evidence showed that the genetic changes and expression disorders of m6A RNA are associated with the tumor occurrence, progression, and treatment resistance. The expression levels of HNRNPC, YTHDC1, ZC3H13, YTHDF2, FTO, YTHDF1, YTHDF3, METTL14, RBM15, WTAP, KIAA1429, FMR1, and HNRNPA2B1 were dramatically higher in UCEC high-risk group (*p* < 0.05) (Supplementary Figure 3A). In addition, the expression levels of mRNAsi (*p* = 3.52e-10) and EREG-mRNAsi (*p* = 0.032) in high-risk group were also higher (Supplementary Figure 3B). To further explore the correlation between FRPS and immune status, we used ssGSEA to quantify the enrichment scores of various immune cell subgroups, related functions, or pathways (Supplementary Figures 3C,D). Interestingly, the antigen presentation process (aDC and iDC) was significantly different, and the enriched cytokine–cytokine receptor interaction in the KEGG analysis scored higher in the high-risk group (*p* < 0.05, Supplementary Figure 3D).

### Gene Set Enrichment Analysis Identifies a Signaling Pathway Related With Ferroptosis-Related Prognosis Signature

In addition, GSEA analyzed the transcription information of patients in the high-risk and low-risk subgroups. Based on normalized *p*-value < 0.05, FDR < 0.05, and NES, we filtered the most significant enrichment biological approach. Representative KEGG pathways were related to some essential signaling pathways including cell cycle, DNA replication, mismatch repair, alpha linolenic acid metabolism, and ribosome and tyrosine metabolism (Supplementary Figure 4). The aforementioned results suggested the potential mechanism of the occurrence and development of UCEC.

### Response to Chemotherapy in Two Groups

Using the pRRophetic algorithm, IC_50_ of 35 common chemotherapeutic agents was predicted in high- or low-risk group (PD.0332991, Nutlin.3a, X17.AAG, Bryostatin.1, PD.0325901, SB.216763, Bicalutamide, AZD6244, PF.02341066, LFM.A13, Temsirolimus, NVP.BEZ235, FTI.277, RDEA119, BMS.536924, MG.132, PF.562271, Roscovitine, AZ628, Vinblastine, EHT.1864, Tipifarnib, BMS.754807, Lapatinib, KIN001.135). All 25 drugs had higher IC_50_ in high-risk patients, indicating that the low-risk patients were more sensitive to these 25 drugs (Wilcoxon test, all *p* < 0.05; Supplementary Figure 5).

### Ferroptosis-Related Prognosis Signature and Consensus Clustering

**FIGURE 9:**
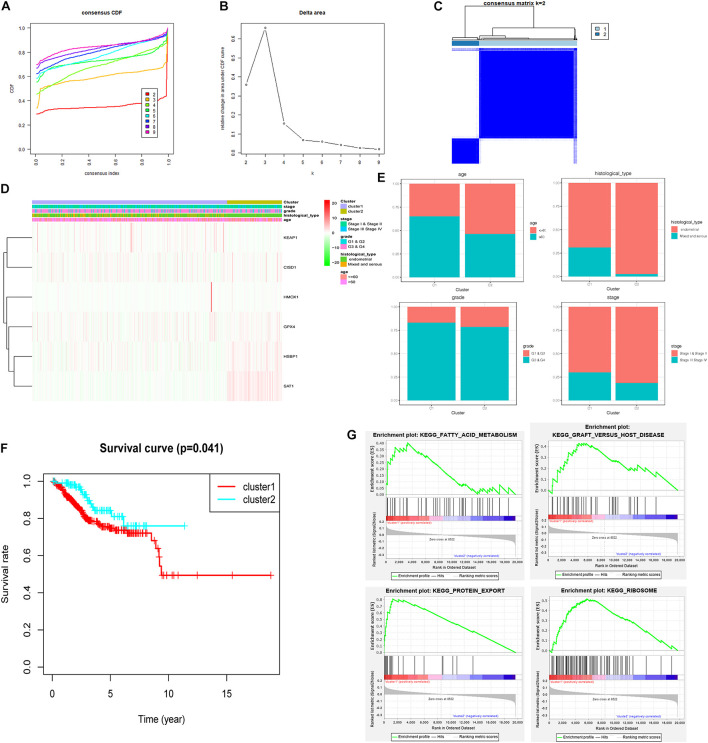
Consensus clustering for ferroptosis-related genes in UCEC patients. **(A)** Consensus clustering CDF for *k* = 2 to *k* = 9. **(B)** Relative change in area under CDF curve for *k* = 2 to *k* = 9. **(C)** Consensus clustering matrix of UCEC samples from TCGA dataset for *k* = 2. **(D)** Heat map of two clusters defined by the six variable expression genes. **(E)** The proportion of clinical factors in two clusters. **(F)** K–M survival curve of patients in two clusters. **(G)** The significantly enriched KEGG pathways in two clusters.

Consensus clustering was analyzed based on the expression levels of six targeted genes. We chose *K* = 2 as the most optimal clustering because the clustering was suboptimal when divided into more than two clusters (Figures 9A–C). UCEC patients in clusters 1 and 2 showed significant differences in age, stage, and histological type, but did not show any significant differences in grade (Figures 9D,E). Moreover, the OS was significantly shorter in the UCEC patients of cluster 1 based on Kaplan–Meier curve compared with those of cluster 2 (Figure 9F). We also compared the significantly enriched KEGG pathways between two clusters; four pathways were identified, including fatty acid metabolism, graft-versus-host disease, protein export, and ribosome. These mechanisms may involve in the pathogenesis of UCEC (Figure 9G).

## Discussion

The incidence rate of uterine corpus endometrial carcinoma (UCEC) is increasing in recent years, becoming a global problem threatening women’s health. To date, therapeutic regimens, such as immunological therapy and chemotherapy, are applied according to the clinical stages of the tumor. However, some patients cannot benefit from the current therapeutic regimens even if they are in the same clinical stage. To overcome this challenge, in this research, we developed a model for predicting the survival and therapeutic response of UCEC patients using ferroptosis-related genes.

Ferroptosis is emerging as an iron-dependent regulation of cell death mediated by the fatal accumulation of lipid peroxides. Previous studies had reported that several genes can serve as regulators of ferroptosis and plays a crucial role in HCC. In this study, we systematically identified the expression of 60 ferroptosis-related genes and then filtered out six prognosis-related genes (HMOX1, KEAP1, HSBP1, SAT1, CISD1, and GPX4) to construct a signature for overall survival prediction in patients with UCEC. The AUC values of the training and testing cohort were greater than 0.7. The FRPS showed a higher prognostic value compared with other clinical factors.

This signature was constructed based on six HMOX1 prognosis-related genes (HMOX1, KEAP1, HSBP1, SAT1, CISD1, and GPX4). HMOX1 is a cell-protective enzyme that is important in maintaining the dynamic balance of REDOX and provides an effective antioxidant defense mechanism in response to cellular stress by breaking down toxic heme into carbon monoxide, biliverdin, and iron (Nath, 1999; Lever et al., 2016). Although HMOX1 expression is upregulated in the process of ferroptosis in cancer cells, it is not clear whether HMOX1 is induced in this context to enhance ferroptosis or as a protective response (Kwon et al., 2015). Based on our results, the imbalance of the expression of HMOX1 suggested the poor prognosis of patients. KEAP1, a component of Nrf2-Keap1 pathway, acts as a molecular switch to activate Nrf2, and KEAP1 senses and delivers the oxidizing challenge (Lau et al., 2008). Nrf2-Keap1 pathway can act as a switch for malignancy in gliomas promoting cell proliferation and resistance to cell death processes such as ferroptosis (Fan et al., 2017). HSBP1 is an evolutionarily conserved heat shock factor binding protein that can directly bind to the DNA of heat shock factor 1 (Hsf1) and inhibit its transcriptional activity (Satyal et al., 1998). There are no large data to prove the function of HSBP1 as a ferroptosis regulator. The transcriptional activation of SAT1 mediated by p53 is essential for ROS-induced ferroptosis because knocking out SAT1 can significantly eliminate the p53-induced ferroptosis under ROS stress (Ou et al., 2016). CISD1, also known as mitoNEET, is an iron-containing mitochondrial outer membrane protein with a size of 13 kDa (Geldenhuys et al., 2014). It was first identified as a target for the treatment of diabetes drug pioglitazone (Colca et al., 2004). Functionally, CISD1 regulates iron uptake and respiration in mitochondria (Wiley et al., 2007; Tamir et al., 2015). CISD1 deficiency leads to iron accumulation and subsequent oxidative damage in mitochondria, which are involved in fat and glucose metabolism (Kusminski et al., 2012). In addition to diabetes, CISD1 expression impairment is also associated with tumor growth (such as breast cancer and liver cancer) and has been considered as a potential chemotherapy target (Salem et al., 2012; Sohn et al., 2013). GPX4 has been determined as a central regulator of ferroptosis (Yang et al., 2014). In models where GPX4 deficiency leads to death or cell loss, iron prolapse is likely to occur. In fact, embryonic fibroblasts (MEF) in conditional Gpx4 knockout mice died of lipid peroxidation after Gpx4 deletion. Supplementation of vitamin E in the medium of the MEF saved cell death (Seiler et al., 2008). In normal cell physiology, the increase in lipid peroxidation caused by GPX4 inhibition raises the question of the origin of lipid peroxidation (Yang and Stockwell, 2008).

We further investigated the biological function of this FRPS. We found this signature was closely related to the tumor immune microenvironment. Several studies had demonstrated that tumor played important role in the developing and prognosing of tumor (Hinshaw and Shevde, 2019). Immune cells are important constituents of the tumor stroma and take part in this process. Immune cells like macrophages M1, macrophages M2, T cell follicular helper, and B cells naive were positively correlated with this signature while NK cells activated, T cells regulatory (Tregs), neutrophils, and dendritic cells resting were negatively correlated with this signature, which hinted the strong immunoreaction in patients from the low-risk group. Apart from the immune cell infiltration, results from the ImmuCellAI showed that patients in the low-risk group may exhibit sensitive response to immunotherapy, thus, may benefit from immunotherapy. This conclusion was also supported by the results from immune checkpoint modulators, TMB, and MSI. Meanwhile, chemotherapy was another important treatment for UCEC patients. According to the estimated IC_50_ results from the GDSC database, the patients in low-risk group were more sensitive to 25 chemotherapy drugs including bicalutamide, temsirolimus, roscovitine, vinblastine, tipifarnib, lapatinib, and other drugs.

However, this research also has several limitations. First, the research is conducted mainly based on online Public Database. To verify our model, the external data were necessary. Second, we mainly focused on the ferroptosis-related genes, and there might be more precise genes which can reflect patient’s prognosis. Finally, the response to chemotherapy was predicted by online database, and some drugs were not the major drugs for UCEC, which should be verified in future search.

## Conclusion

In summary, based on six selected ferroptosis-related genes, we constructed a prognostic signature possessing independent predictive value of UCEC patients in TCGA datasets. Through internal verification, the versatility of the signature was proven and the nomogram was showed to be suitable for clinical use. With the help of FRPS, clinical factors can predict a patient’s response to immunotherapy and chemotherapy, which can provide valuable information on designing a therapeutic regimen.

## Data Availability Statement

The datasets presented in this study can be found in online repositories. The names of the repository/repositories and accession number(s) can be found in the article/Supplementary Material.

## Ethics Statement

The studies involving human participants were reviewed and approved by the Affiliated Tumor Hospital to Nantong University. The patients/participants provided their written informed consent to participate in this study. Written informed consent was not obtained from the individual(s) for the publication of any potentially identifiable images or data included in this article.

## Author Contributions

TN and HZ conceived the study. JL, YW, HM, and YY participated in the design, analysis, and draft of the study. JL and YW plotted all figures in this article. HM and YY helped in the data analysis. All authors approved the final version of this article and agreed to be accountable for all aspects of the work.

## Conflict of Interest

The authors declare that the research was conducted in the absence of any commercial or financial relationships that could be construed as a potential conflict of interest.

## Publisher’s Note

All claims expressed in this article are solely those of the authors and do not necessarily represent those of their affiliated organizations, or those of the publisher, the editors and the reviewers. Any product that may be evaluated in this article, or claim that may be made by its manufacturer, is not guaranteed or endorsed by the publisher.

## Footnotes

1
http://cancergenome.nih.gov/

2
https://www.cancerrxgene.org

## Supplementary Material

Supplementary Figure 1 The mRNA expression level of **(A)** CISD1, GPX4, HSBP1, SAT1, and their **(B)** Kaplan–Meier plot. ***P* < 0.01 and ****P* < 0.001.

Supplementary Figure 2 Kaplan–Meier plot of the high- and low-risk groups under diverse situations by classifying the patients into different subgroups according to **(A)** age, **(B)** tumor grade, **(C)** tumor pathological pattern, and **(D)** stage.

Supplementary Figure 3 m6A regulators, mRNAsi, and functional analyses in two groups. **(A)** The expression levels of HNRNPC, YTHDC1, ZC3H13, YTHDF2, FTO, YTHDF1, YTHDF3, METTL14, RBM15, WTAP, KIAA1429, FMR1, and HNRNPA2B1 were dramatically higher in UCEC high-risk group than in low-risk group. **(B)** The expression levels of mRNAsi and EREG-mRNAsi in high-risk and low-risk groups. **(C)** The correlation between the FRPS and scores of 16 immune cells determined by ssGSEA. **(D)** The enriched cytokine–cytokine receptor interaction determined by ssGSEA. The low-risk and high-risk groups arerepresented via blue and red violin, respectively.

Supplementary Figure 4 Significantly enriched KEGG pathway related to FRPS identified by GSEA analysis.

Supplementary Figure 5 The predicted chemotherapeutic response in the high-risk and low-risk groups.

Supplementary Table 1 The basic clinical information of the involved patients in TCGA.

Supplementary Table 2 Primers used in PCR application.

Supplementary Table 3 The correlation between the signature and clinical factors.
