# Supplementary figures and images for: Identification of the Prognostic Signature Associated With Tumor Immune Microenvironment of Uterine Corpus Endometrial Carcinoma Based on Ferroptosis-Related Genes
Source: Front Cell Dev Biol. 2021 Oct 6;9:735013. doi: 10.3389/fcell.2021.735013 (PMC8526722; doi:10.3389/fcell.2021.735013)

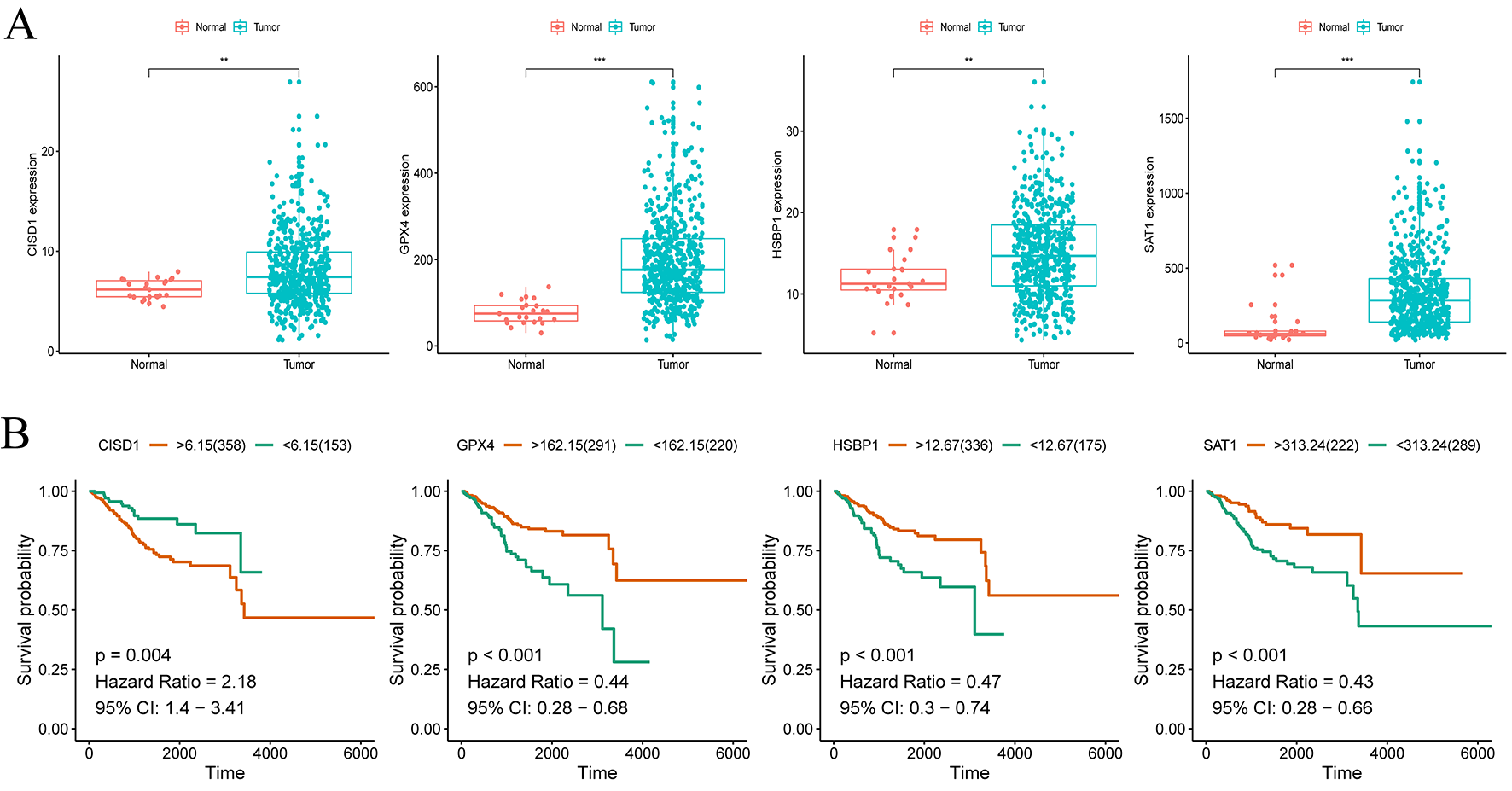

Supplement: Supplementary Figure 1 — The mRNA expression level of (A) CISD1, GPX4, HSBP1, SAT1, and their (B) Kaplan–Meier plot. **P < 0.01 and ***P < 0.001. [file Image_1.TIF]

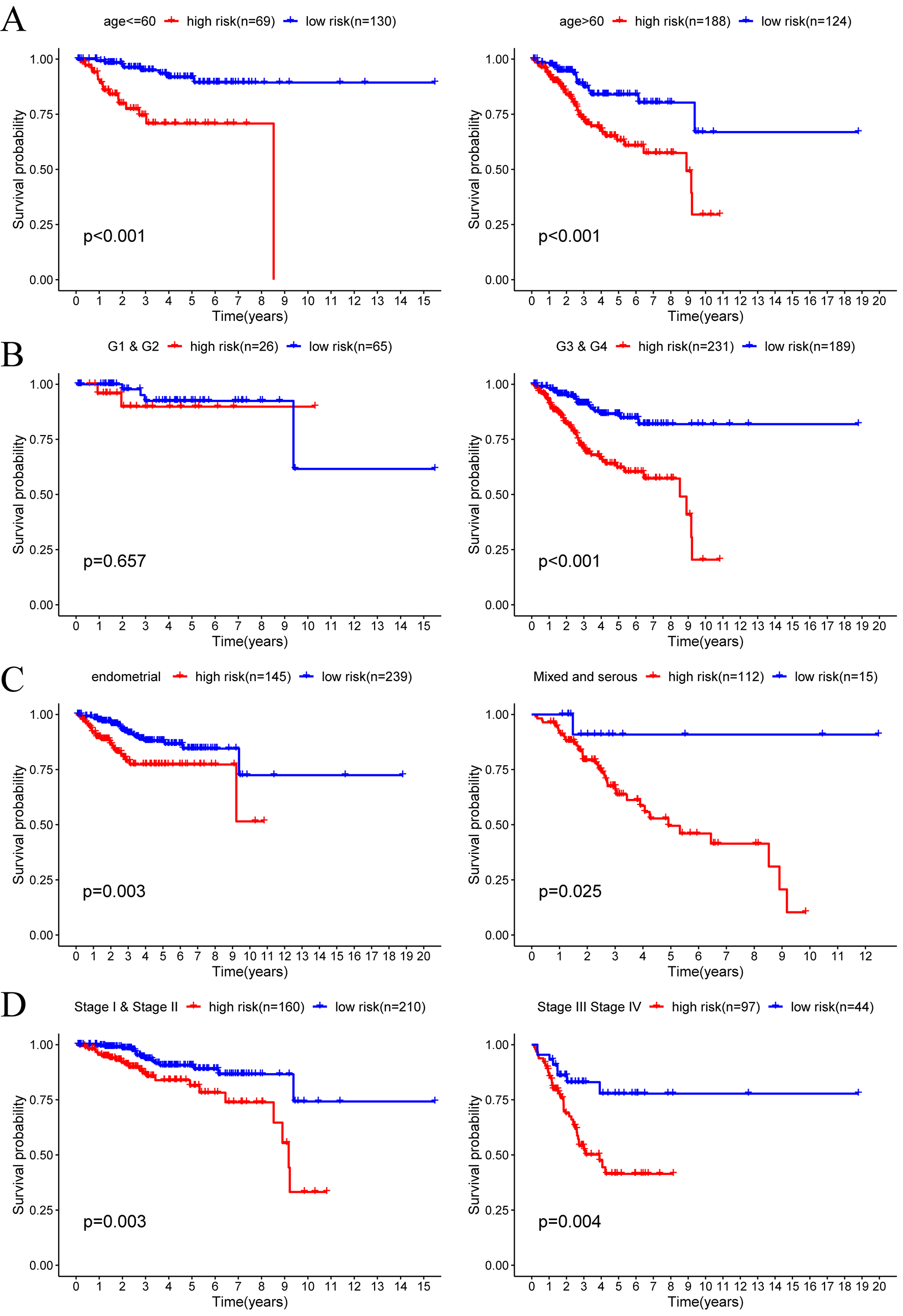

Supplement: Supplementary Figure 2 — Kaplan–Meier plot of the high- and low-risk groups under diverse situations by classifying the patients into different subgroups according to (A) age, (B) tumor grade, (C) tumor pathological pattern, and (D) stage. [file Image_2.TIF]

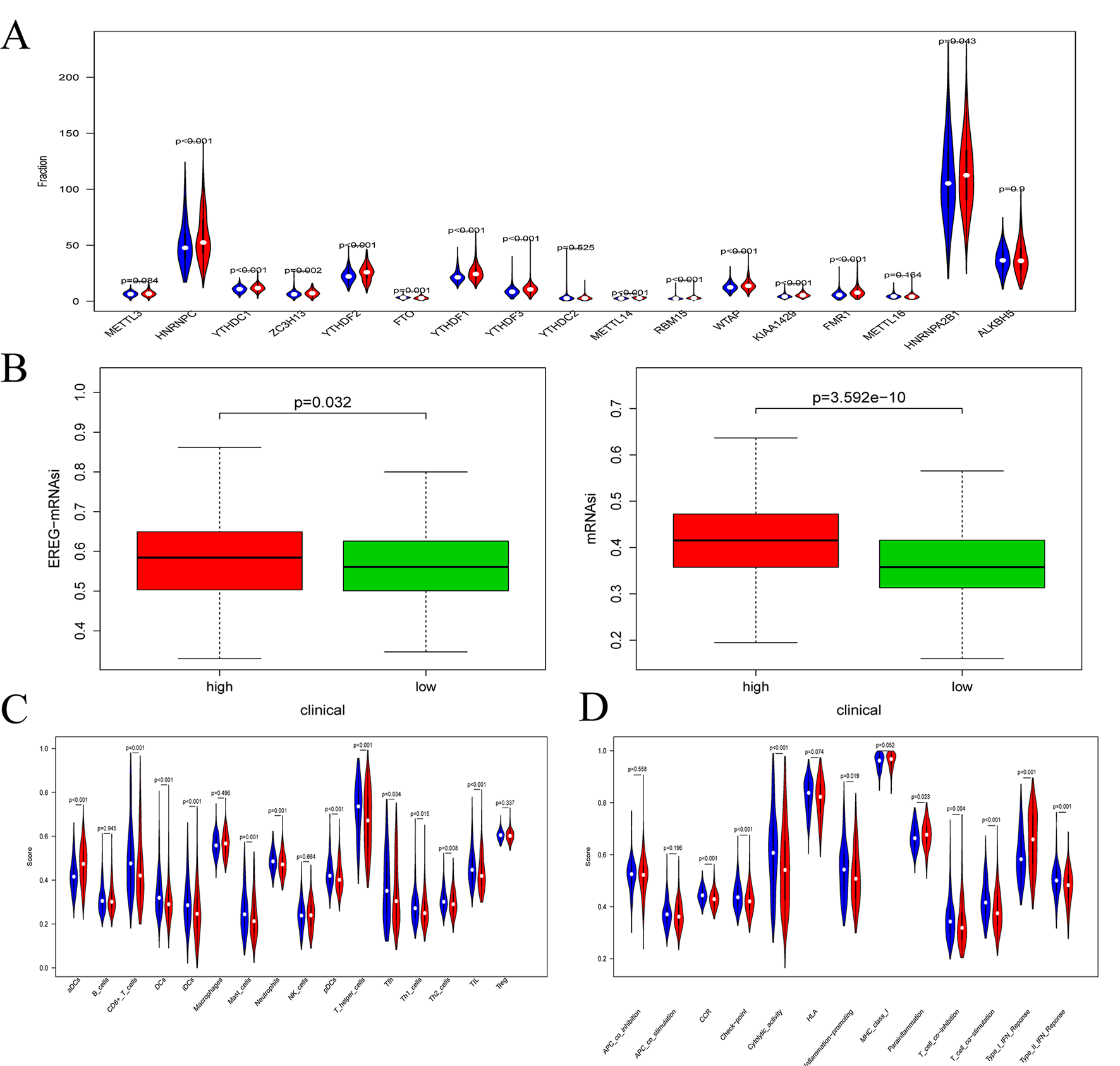

Supplement: Supplementary Figure 3 — m6A regulators, mRNAsi, and functional analyses in two groups. (A) The expression levels of HNRNPC, YTHDC1, ZC3H13, YTHDF2, FTO, YTHDF1, YTHDF3, METTL14, RBM15, WTAP, KIAA1429, FMR1, and HNRNPA2B1 were dramatically higher in UCEC high-risk group than in low-risk group. (B) The expression levels of mRNAsi and EREG-mRNAsi in high-risk and low-risk groups. (C) The correlation between the FRPS and scores of 16 immune cells determined by ssGSEA. (D) The enriched cytokine–cytokine receptor interaction determined by ssGSEA. The low-risk and high-risk groups arerepresented via blue and red violin, respectively. [file Image_3.TIF]

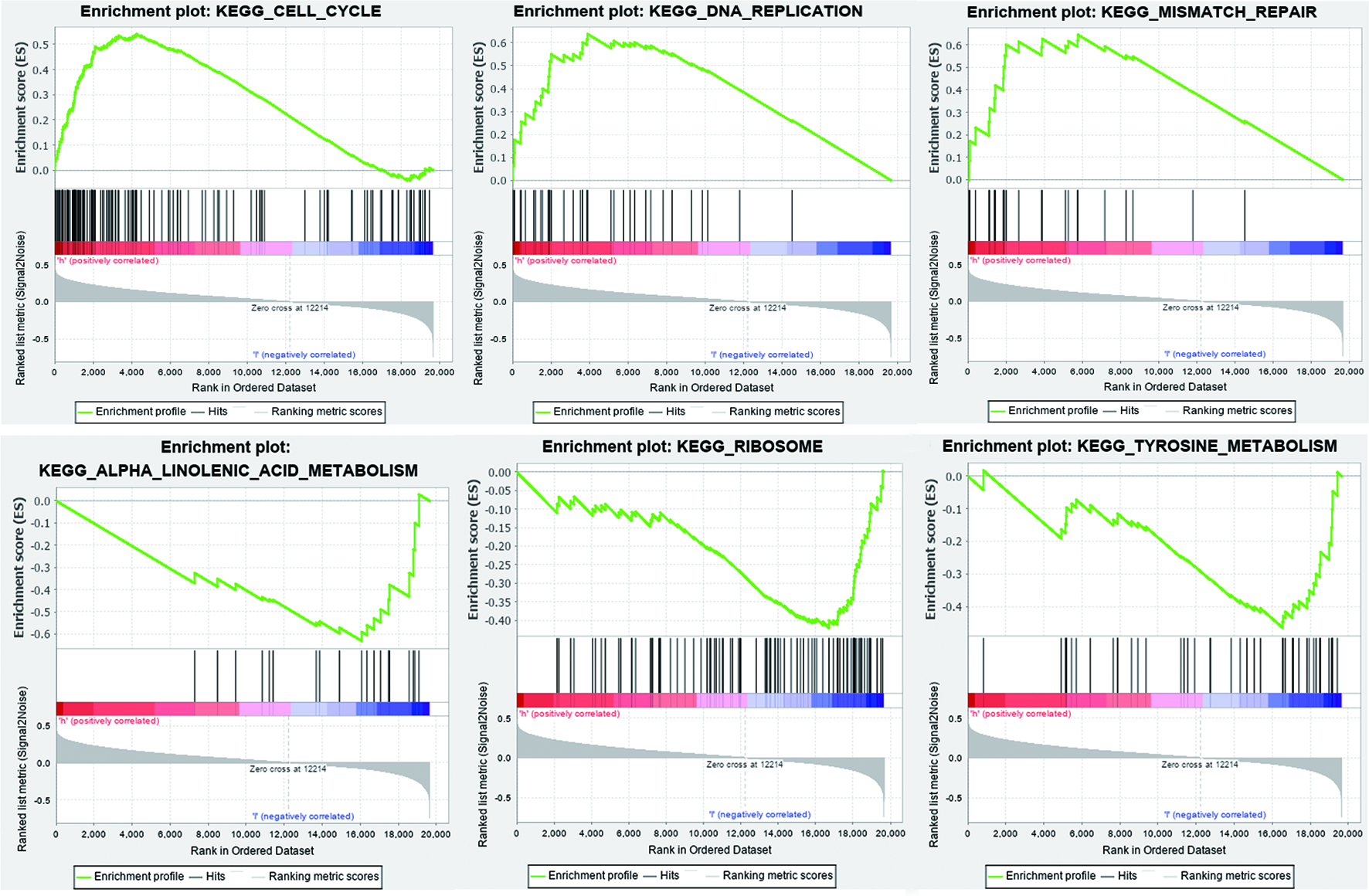

Supplement: Supplementary Figure 4 — Significantly enriched KEGG pathway related to FRPS identified by GSEA analysis. [file Image_4.TIF]

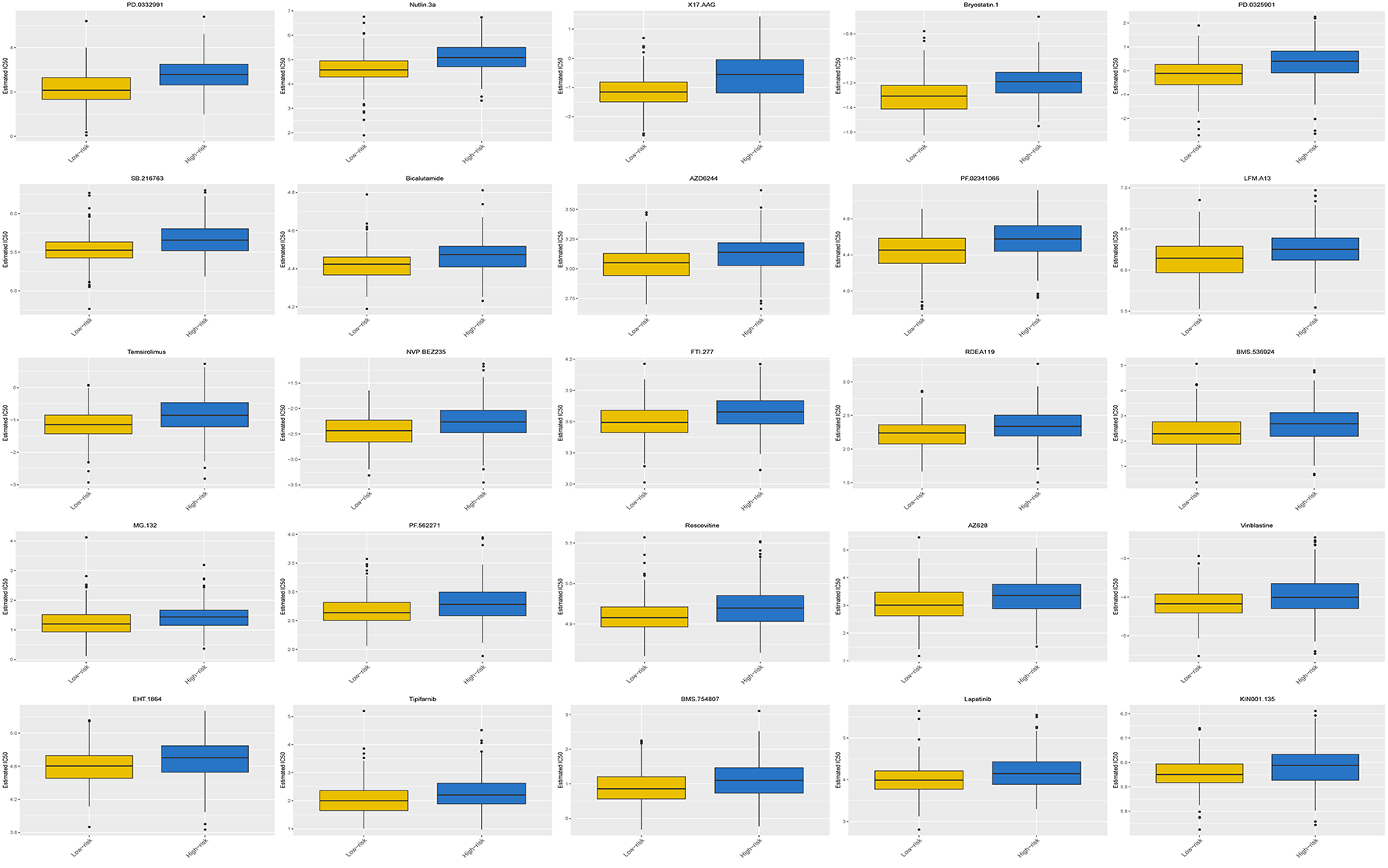

Supplement: Supplementary Figure 5 — The predicted chemotherapeutic response in the high-risk and low-risk groups. [file Image_5.TIF]
