# Supplementary material for: Identification of the Prognostic Signature Associated With Tumor Immune Microenvironment of Uterine Corpus Endometrial Carcinoma Based on Ferroptosis-Related Genes
Source: Front Cell Dev Biol. 2021 Oct 6;9:735013. doi: 10.3389/fcell.2021.735013 (PMC8526722; doi:10.3389/fcell.2021.735013)
Supplement: Supplementary Table 1 — The basic clinical information of the involved patients in TCGA. [file Table_1.docx]

Table S1. The basic clinical information of the involved patients

| Covariates | Type | Total set (n=511) | Train set (n=256) |
| --- | --- | --- | --- |
| Age, no (%) | ＜=60 | 199(38.94%) | 102(39.84%) |
|  | ＞60 | 312(61.06%) | 154(60.16%) |
| Histological type, no (%) | Endometrial | 384(75.15%) | 185(72.27%) |
|  | Mixed and serous | 127(24.85%) | 71(27.73%) |
| Grade, no (%) | G1 & G2 | 91(17.81%) | 46(17.97%) |
|  | G3 & G4 | 420(82.19%) | 210(82.03%) |
| Stage, no (%) | Stage I & Stage II | 370(72.41%) | 187(73.05%) |
|  | Stage III Stage IV | 141(27.59%) | 69(26.95%) |
| Survival status (%) | Alive | 422(82.58%) | 214(83.59%) |
|  | Dead | 89(17.42%) | 42(16.41%) |
