# Supplementary material for: Identification of the Prognostic Signature Associated With Tumor Immune Microenvironment of Uterine Corpus Endometrial Carcinoma Based on Ferroptosis-Related Genes
Source: Front Cell Dev Biol. 2021 Oct 6;9:735013. doi: 10.3389/fcell.2021.735013 (PMC8526722; doi:10.3389/fcell.2021.735013)
Supplement: Supplementary Table 2 — Primers used in PCR application. [file Table_2.docx]

Table S2. Primers used in PCR application

| Gene | Forward primers | Reverse primer |
| --- | --- | --- |
| HMOX1 | AAGACTGCGTTCCTGCTCAAC | AAAGCCCTACAGCAACTGTCG |
| KEAP1 | CTGGAGGATCATACCAAGCAGG | GGATACCCTCAATGGACACCAC |
| HSBP1 | ATCTGGAAAAGAATATCGCGGAC | TTGCGTGGCAGGTATCTTGTT |
| SAT1 | ACCCGTGGATTGGCAAGTTAT | TGCAACCTGGCTTAGATTCTTC |
| CISD1 | GATCGCAGCAGTTACCATTGC | GCATGTACTATCTTGGGGTTGTC |
| GPX4 | GAGGCAAGACCGAAGTAAACTAC | CCGAACTGGTTACACGGGAA |
