# Supplementary material for: Identification of the Prognostic Signature Associated With Tumor Immune Microenvironment of Uterine Corpus Endometrial Carcinoma Based on Ferroptosis-Related Genes
Source: Front Cell Dev Biol. 2021 Oct 6;9:735013. doi: 10.3389/fcell.2021.735013 (PMC8526722; doi:10.3389/fcell.2021.735013)
Supplement: Supplementary Table 3 — The correlation between the signature and clinical factors. [file Table_3.docx]

Table S3. The correlation between the signature and clinical factors

| Gene id | Age | Histological type | Grade | Stage |
| --- | --- | --- | --- | --- |
| HMOX1 | -1.088(0.278) | 1.032(0.303) | -1.908(0.057) | -1.168(0.245) |
| KEAP1 | 1.03(0.304) | -4.124(6.394e-05) | -4.63(5.821e-06) | -1.657(0.099) |
| HSBP1 | -4.26(2.483e-05) | 10.046(3.394e-20) | 3.396(8.839e-04) | 3.364(8.808e-04) |
| SAT1 | -4.452(1.109e-05) | 15.001(9.332e-42) | 3.972(1.186e-04) | 5.29(2.301e-07) |
| CISD1 | 1.024(0.306) | -1.127(0.261) | -3.459(7.042e-04) | -2.255(0.025) |
| GPX4 | -2.193(0.029) | 5.314(2.791e-07) | 0.667(0.506) | 2.349(0.020) |
| FRPS | -0.235(0.814) | -3.086(0.002) | -3.232(0.001) | -1.676(0.096) |
